# Antimicrobial Usage Monitoring Systems and Stewardship of Antimicrobials in Animal Health

**DOI:** 10.3390/ani14223325

**Published:** 2024-11-19

**Authors:** Carla Miranda

**Affiliations:** 1 UCIBIO, Applied Molecular Biosciences Unit, University Institute of Health Sciences (1H-TOXRUN, IUCS-CESPU), 4585-116 Gandra, Portugal; carla.miranda@iucs.cespu.pt; 2 LAQV-REQUIMTE–Associated Laboratory for Green Chemistry, University NOVA of Lisbon, 1099-085 Caparica, Portugal

Antimicrobials, especially antibiotics, have transformed modern medicine; significantly impacted the health of humans, animals, and plants; and enhanced food safety and security. The initial results in the treatment of infectious diseases were extraordinary. However, the excessive and uncontrolled use of antimicrobials in human and veterinary medicine and agriculture has created a catastrophic public health issue, hindering the control and treatment of infectious diseases. The inappropriate and overuse of antibiotics expose bacteria to selective pressure, making it the primary factor in the emergence of new drug-resistant bacteria, including multidrug-resistant (≥3 antimicrobial classes) and pandrug-resistant (all current antimicrobial classes) microorganisms. This global health emergency, highlighted by the World Health Organization (WHO), contributes to millions of deaths each year. Recent studies suggest that by 2050, if sufficient action is not taken to address the threat of antimicrobial resistance, it could cause the death of approximately 10 million people globally [1].

With the advancement of knowledge of and diagnostic techniques for detecting antimicrobial resistance (AMR), the role of therapeutic drug monitoring and antimicrobial stewardship—focused on optimizing therapy and reducing antibiotic use—has gained prominence in recent years. This shift has led to discussions and revisions of current clinical practices and drug dosages, stricter oversight of professionals authorized to administer these drugs, and tighter regulation of their sale and the tracking of quantities. Moreover, the WHO Global Action Plan on Antimicrobial Resistance, a global approach to overcoming this problem, is supported by the WHO, the World Organization for Animal Health, the Food and Agriculture Organization of the United Nations, and the UN Environment Program, who collectively signed a memorandum of understanding on antimicrobial resistance in March 2023, promoting a ‘One Health’ strategy [2]. The ‘One Health’ concept stresses the interconnectedness of human, animal, and environmental health, emphasizing that interactions and exchanges occur among them.

In animals, antimicrobials are used for various purposes, including treatment, prevention, and control, and to enhance performance and welfare as growth promoters. Despite the continued use of growth promoters in some countries outside the European Union, their use has been decreasing sharply. The implementation of various multidisciplinary measures showed a decrease of around 13% in global antimicrobial use in animals between 2017 and 2019, but also a decline in antimicrobials used in animals classified as being of highest priority and critical importance for human health, representing < 20% in 2019; this is estimated to be the best indicator for the global monitoring of antimicrobials [3]. In addition, there was a 44% reduction in total antimicrobial consumption in food-producing animals between 2014 and 2021, while at the human level, their consumption remained stable [4]. However, it is projected that antibiotic consumption in livestock production will double between 2010 and 2030 in some countries [3,4]. Additionally, the online sale of veterinary antibiotics without a prescription has increased, and there are limited data on antimicrobial use in pets. These factors may contribute to a rise in microorganisms with resistance genes in animals. Consequently, antimicrobial resistance in bacteria from pets and livestock can be transmitted to humans through close contact, the food chain, or the environment, reaching all sectors.

This Special Issue, entitled Antimicrobial Usage Monitoring Systems and Stewardship of Antimicrobials in Animal Health, aims to encourage the publication of original research papers and reviews on the impact of antimicrobial usage on animal health, including the design and implementation of cross-sectional surveys, stewardship programs, and measures for the monitoring, control, and mitigation of antimicrobials, as well as measures for tackling antibiotic resistance and the development of new antibiotic alternatives.

This Special Issue includes eight research papers: seven original manuscripts and one communication. These papers highlight several crucial aspects of the impact of antimicrobial usage on animal health, including the following:

(i) Monitoring antimicrobial usage in animals;
(ii) The impact of antibiotic stewardship interventions on antibiotic administration by veterinarians;
(iii) Monitoring and detecting the occurrence of AMR in bacteria from various sources, such as sick broilers, colostrum, and ornamental animal feed;
(iv) Screening new agents with antibacterial properties for potential use in combination with, or as alternatives to, antibiotics.

Based on the data collected by an organized survey targeting reliable government and non-government organizations, Upadhyaya et al. [5] reported features of antimicrobial usage in food-producing animals in Nepal for three years (2018–2020), showing a sharp decline in the production and importation of the active ingredients of antimicrobials and of critical antibiotics. The most used antibiotics were oxytetracycline parenterally, tilmicosin orally, and sulfadimidine both parenterally and orally, all with therapeutic proposes. This study reflects on the effectiveness of the prudent use of antibiotics, as well as the efforts made and mitigation strategies proposed to overcome their overuse, contributing to our knowledge of the trend of national antimicrobial use and enabling better planning and more effective interpretation of resistance surveillance data. Jerab et al. [6] quantify and compare antimicrobial usage in two distinct broiler hatchery systems: on-farm and traditional hatching. Using the new method, on-farm hatching, in which fertilized broiler eggs are transported to farms at 17–19 incubation days, demonstrated more antimicrobial-free flocks with a lower antimicrobial treatment incidence, and consequently, a 5.6 times lower probability of antimicrobial use than the traditional method. These results suggest that on-farm broiler production could represent an alternative system to mitigate antimicrobial use.

Acharya et al. [7] addressed the impact of an antibiotic stewardship intervention system on antibiotic prescribing among veterinarians in two countries, Canada and Israel, in which antibiotic prescribing guidelines were provided for veterinarian participants. This approach is strongly advised in veterinary clinics to minimize the unnecessary use of antibiotics, particularly the administration of critical antibiotics. It has been demonstrated that antibiotic stewardship interventions enhance antibiotic prescribing practices in a veterinary context.

The following three articles focused on detecting antimicrobial resistance in significant bacteria, *Enterococcus* and *Escherichia coli*. One of these studies revealed a high prevalence of multidrug-resistant *Enterococcus* (92%) in bovine colostrum [8]. Bovine colostrum may serve as a significant reservoir for bacteria and a vehicle for spreading antibiotic resistance and virulence genes across the three niches within the One Health framework. This underscores the critical need for stringent hygiene and sanitary practices to reduce microbial contamination in colostrum. Most of the *Enterococcus* isolates showed phenotypic resistance to quinupristin-dalfopristin, erythromycin, tetracycline, and streptomycin. This resistance was confirmed by the identification of the resistance genes *tetK*, *tetM*, and *tetL* (for tetracycline), *ermB* (for macrolides), and *ant(6)-Ia* (for aminoglycosides). The most frequently detected virulence factors were *cpd*, *esp*, *agg*, and *cyl*L_L_ [8]. *Enterococcus* isolates obtained from ornamental animal feed showed 48% multidrug-resistant isolates, and most exhibited high resistance to rifampicin and erythromycin [9]. Most isolates carried the resistance genes *erm*B and *tet*L, along with the virulence genes *cyl*L_L_ and *esp*. Among these species, *Enterococcus gallinarum* had the highest number of multidrug-resistant isolates and virulence genes compared to *E. faecalis* and *E. faecium*. These results underscore the high levels of antibiotic-resistant *Enterococcus* species found in ornamental animal feed and raise concerns about the increasing interaction between these animals and humans as a public health issue [9]. Additionally, [10] employed a genomic approach to analyze AMR in extraintestinal *E. coli* during the enrofloxacin treatment of broilers with colisepticemia. The results showed an increase in population diversity after treatment, and plasmid-mediated fluoroquinolone resistance was neither disseminated nor persistent among the observed genomes, unlike plasmids carrying other AMR genes, both before and after treatment. The study discusses the persistence of plasmids not linked to antimicrobial selective pressure, suggesting that further research is needed to confirm these findings.

This Special Issue also includes two studies [11,12] on antimicrobial alternatives to reduce antibiotic use. One study investigated new antimicrobials against *Salmonella*, using acidulants to coat dry pet food kibbles containing ingredients like canola oil, chicken fat, Menhaden fish oil, tallow, and lard. Adding acidulants, such as sodium bisulfate and organic acids, during coating with fats and oils showed promising potential in controlling *Salmonella* present in the fat or oil system within 2 h [11]. Another study reported new potential alternative antimicrobials against multidrug-resistant *Salmonella enterica*, using cinnamon, clove, oregano, and red thyme in combination with oxytetracycline. Although the positivity of the interaction depended on the bacterial strain, synergy and additivity were observed between the four tested oils and oxytetracycline. This suggests that, in particular, the combination of cinnamon oil with oxytetracycline could be an effective alternative to mitigate tetracycline-resistant *Salmonella* [12].

This Special Issue consolidates multidisciplinary research on antimicrobial monitoring and stewardship in animals. Identifying gaps in control and registration systems, along with the continuous monitoring and surveillance of antimicrobial use in animals, is crucial for improving antimicrobial stewardship and enhancing measures to prevent the overuse of antimicrobials on a global scale, such as vaccination, better hygiene, or alternative drugs. To control AMR bacteria in animals and their products, investigating and detecting them is crucial to understanding their global dissemination. This knowledge is essential for discussing and screening new agents as effective alternatives to current antimicrobials or for combining them to reduce dosages or toxicity effects.
